# Interleukin-1 receptor type 1 is overexpressed in neurons but not in glial cells within the rat superficial spinal dorsal horn in complete Freund adjuvant-induced inflammatory pain

**DOI:** 10.1186/s12974-017-0902-x

**Published:** 2017-06-23

**Authors:** Krisztina Holló, László Ducza, Zoltán Hegyi, Klaudia Dócs, Krisztina Hegedűs, Erzsébet Bakk, Ildikó Papp, Gréta Kis, Zoltán Mészár, Zsuzsanna Bardóczi, Miklós Antal

**Affiliations:** 1 Department of Anatomy, Histology and Embryology, Faculty of Medicine, University of Debrecen, Nagyerdei krt. 98, 4012 Debrecen, Hungary; 2 Laboratory of Endocrine Neurobiology, Institute of Experimental Medicine, Hungarian Academy of Sciences, Budapest, Hungary; 3 Department of Anatomy, Histology and Embryology, Faculty of Pharmacy, University of Debrecen, Nagyerdei krt. 98, 4012 Debrecen, Hungary; 4 MTA-DE Neuroscience Research Group, Nagyerdei krt. 98, 4012 Debrecen, Hungary

**Keywords:** IL-1R1, Superficial spinal dorsal horn, Rodents, Inflammatory pain evoked by CFA injection, Immunohistochemistry

## Abstract

**Background:**

All known biological functions of the pro-inflammatory cytokine interleukin-1β (IL-1β) are mediated by type 1 interleukin receptor (IL-1R1). IL-1β–IL-1R1 signaling modulates various neuronal functions including spinal pain processing. Although the role of IL-1β in pain processing is generally accepted, there is a discussion in the literature whether IL-1β exerts its effect on spinal pain processing by activating neuronal or glial IL-1R1. To contribute to this debate, here we investigated the expression and cellular distribution of IL-1R1 in the superficial spinal dorsal horn in control animals and also in inflammatory pain.

**Methods:**

Experiments were performed on rats and wild type as well as IL-1R1-deficient mice. Inflammatory pain was evoked by unilateral intraplantar injection of complete Freund adjuvant (CFA). The nociceptive responsiveness of control and CFA-treated animals were tested daily for withdrawal responses to mechanical and thermal stimuli before and after CFA injection. Changes in the expression of 48 selected genes/mRNAs and in the quantity of IL-1R1 protein during the first 3 days after CFA injection were measured with the TaqMan low-density array method and Western blot analysis, respectively. The cellular localization of IL-1R1 protein was investigated with single and double staining immunocytochemical methods.

**Results:**

We found a six times and two times increase in IL-1R1 mRNA and protein levels, respectively, in the dorsal horn of CFA-injected animals 3 days after CFA injection, at the time of the summit of mechanical and thermal allodynia. Studying the cellular distribution of IL-1R1, we found an abundant expression of IL-1R1 on the somatodendritic compartment of neurons and an enrichment of the receptor in the postsynaptic membranes of some excitatory synapses. In contrast to the robust neuronal localization, we observed only a moderate expression of IL-1R1 on astrocytes and a negligible one on microglial cells. CFA injection into the hind paw caused a remarkable increase in the expression of IL-1R1 in neurons, but did not alter the glial expression of the receptor.

**Conclusion:**

The results suggest that IL-1β exerts its effect on spinal pain processing primarily through neuronal IL-1R1, but it can also interact in some extent with IL-1R1 expressed by astrocytes.

## Background

Interleukin-1β (IL-1β) is a major pro-inflammatory cytokine, an inflammatory mediator of peripheral immune responses [1]. In recent years, it has become clear that IL-1β, in association with activated glial cells, modulate also neuronal functions including spinal pain processing [2–5]. Glial cell-produced IL-1β facilitates transmission of noxious inputs at the spinal level [4, 6] and becomes upregulated in the spinal cord under different chronic pain conditions like inflammatory [4, 7–10], neuropathic [11–13], or bone cancer pain [14]. Intrathecal IL-1β administration induces mechanical and thermal hyperalgesia as well as allodynia [15–21]. IL-1β released from astrocytes facilitates excitatory synaptic transmission in the spinal dorsal horn [15, 21–25] by enhancing AMPA and NMDA receptor-mediated increase of inward current and intracellular Ca^2+^ concentration [9, 26–30]. In addition, IL-1β also produces long-term neuronal plasticity in the pain circuits by inducing the phosphorylation of the transcription factor cAMP-response element binding protein [21, 31].

The biological effects of IL-1β are mediated by two distinct receptors. The p80 or type 1 interleukin receptor (IL-1R1), which mediates all of the known biological effects of IL-1β [32], appears to be the functional form of the receptor [33, 34]. The p68 or type 2 interleukin receptor (IL-1R2), which has only a short 29-amino acid cytoplasmic domain, has been identified as a non-functional “decoy” receptor [35, 36]. Upon binding of IL-1β to IL-1R1, an additional protein, termed IL-1 receptor accessory protein gets recruited at the cell membrane to form a high-affinity binding receptor complex with IL-1R1 leading to intracellular signaling [37, 38].

IL-1R1 has been localized widely in the central nervous system including the spinal cord [7, 9, 28, 39]. There is strong evidence that IL-1R1 is expressed in neurons [9, 13, 28, 40]. Concerning its sub-cellular localization, IL-1R1 is distributed along dendrites and enriched in postsynaptic membranes of excitatory synapses, as shown by the high degree of co-precipitation with postsynaptic density protein PSD-95 and NR2B subunit of NMDA receptors [41]. Other reports, however, suggest that IL-1R1 may be expressed not only in neurons but also in glial cells [13, 28, 42–45] including astrocytes, activated microglia, oligodendrocytes, and ependymal cells [43, 46].

According to the prevailing view, the synaptic effects of IL-1β are mediated by direct actions on neurons [47–49]. In the superficial spinal dorsal horn, however, Gruber-Schoffnegger et al. [45] reported that the synaptic effects of IL-1β were not mediated directly via activation of neuronal IL-1R1, but rather indirectly via IL-1R1 being expressed on glial cells. According to this alternative signaling pathway, IL-1β primarily acts on glial IL-1R1. Activation of glial cells leads to the release of further cytokines, chemokins, and gliotransmitters [50, 51] which may amplify glutamatergic synaptic transmission triggering long-term potentiation (LTP) at C-fiber synapses and hyperalgesia [45]. Because of this contradiction in the interpretation of the effects of IL-1β on nociceptive information processing, here we intended to reinvestigate the expression and cellular distribution of IL-1R1 in the superficial spinal dorsal horn. We intended to explore the expression pattern of IL-1R1 on neurons and glial cells in control animals and also in Freund adjuvant-evoked inflammatory pain.

## Methods

### Animals

Experiments were performed on male Wistar rats (Gödöllő, Hungary) and wild type as well as IL-1R1-deficient mice (B6.129S7-Il1r1 ^tm1/mx^,/J stock # :003245) created by Labow et. al. (1997) and purchased from Jackson Laboratories (Bar Harbor, ME, USA). All animal study protocols were approved by the Animal Care and Protection Committee at the University of Debrecen and were carried out in accordance with the European Community Council Directives. Animals were housed individually in a temperature-controlled (24 °C) colony room and maintained on a 12 h/12 h light/dark cycle. Food and water were provided ad libitum. Animals were divided into three experimental groups: Experimental group 1 (15 rats, 6 wild type, and 6 IL-1R1-deficient mice): injection of 100 and 50 μl complete Freund-adjuvant (CFA) into the plantar surface of the right hind paw of rats and mice, respectively. Experimental group 2 (9 rats, 6 wild type, and 6 IL-1R1-deficient mice): injection of 100 and 50 μl physiological saline into the plantar surface of the right hind paw of rats and mice, respectively. Experimental group 3 (15 rats, 6 wild type, and 6 IL-1R1 deficient mice): animals without any treatment were used as controls.

### Behavioral tests

All animals were tested for withdrawal responses to noxious mechanical and thermal stimuli using a dynamic plantar aestesiometer (Ugo Basile, Comerio, VA, Italy) and a Plantar Test Instrument—Hargreaves Apparatus (Ugo Basile, Comerio, VA, Italy), respectively. The mechanical withdrawal threshold (MWT) and the thermal withdrawal latency (TWL) for both hind paws were measured before CFA or saline injection, and the tests were repeated daily after CFA injection. On the six animals in each experimental groups which were used only for behavioral testing, the measurements were carried out till post-injection day 11, whereas on animals which were used for other experiments, MWT and TWL were measured only until post-injection day 3, when the summit of mechanical and thermal sensitivities were detected.

#### Measurement of mechanical sensitivity

Animals were placed upon a network platform and covered with a Perspex enclosure that rendered the animals unrestrained for the duration of the measurement. The hind paw of the animal was positioned above a von Frey-type filament with a tip diameter of about 0.5 mm. The filament exerted an increasing pressure to the plantar surface until the animal removed its paw. At this point, the measurement was terminated and the actual force at which paw withdrawal occurred was recorded. The measurement was repeated five times in each case. From the 30 experimental data (six animals in each group and five measurements on each hind paw), the mean value and standard error of mean (SEM) were calculated. Statistical differences among the data were calculated according to the Kruskal-Wallis test.

#### Measurement of thermal sensitivity

Animals were placed upon a plastic platform and covered with a Perspex enclosure that rendered the animals unrestrained for the duration of the measurement. The hind paw of the animal was positioned above an infrared light source which was focused onto the plantar surface. The illumination which exerted a heat stimulus to the plantar surface was applied until the animal removed its paw. At this point the measurement was terminated and the actual time elapsed until the withdrawal of the paw was recorded. The measurements were repeated five times in each case. From the 30 experimental data (six animals in each group and five measurements on each hind paw), the mean values and standard error of means were calculated. Statistical differences among the data were calculated according to the Kruskal-Wallis test.

### TaqMan low-density array

After the exposure of the spinal cord with serial laminectomy, the L3–L5 lumbar segments of the spinal cord were dissected out from three rats in each experimental group 3 days after CFA injection. The dorsal horn ipsilateral to the site of injection was separated from the rest of the spinal cord and was kept for further processing. Immediately after tissue removal RNA was isolated (RNeasy Lipid tissue kit, Qiagen, Maryland, USA), and cDNA library was created (High Capacity cDNA Reverse Transcription Kit, Applied Biosystems, Foster City, USA, catalog no.: 4368814). RNA quality was tested by Agilent microelectrophoresis system and by Nanodrop photometric analysis. One hundred monograms of cDNA was loaded on each port of the custom designed TaqMan low-density array card (TLDA; Applied Biosystems, Foster City, USA) containing 48 gene format including the gene encoding IL-1R1 and three housekeeping as well as one mandatory control gene (the list of genes is shown in Table 1). PCR reactions were performed by means of ABIPrism7000 real-time PCR system (Applied Biosystems, catalog no.: 4328895). The ABI TaqMan sodium dodecyl sulfate (SDS) software was utilized to obtain the threshold cycle (Ct) values. Data have been obtained from three separate experiments.

**Table 1:** List of genes the RNA transcripts of which were tested with the TaqMan Low Density Array

#	Gene symbol	Full name	Assay ID
1	Grin1	Ionotropic glutamate receptor NMDA1	Grin1-Rn00433800_m1
2	Grin2a	Ionotropic glutamate receptor NMDA2A	Grin2a-Rn00561341_m1
3	Grin2c	Ionotropic glutamate receptor NMDA2C	Grin2c-Rn00561364_m1
4	Gria2	Ionotropic glutamate receptor, AMPA2	Gria2-Rn00568514_m1
5	Gria3	Ionotropic glutamate receptor, AMPA3	Gria3-Rn00583547_m1
6	Gria4	Ionotropic glutamate receptor, AMPA4	Gria4-Rn00568544_m1
7	Grm5	Metabotropic glutamate receptor 5	Grm5-Rn00566628_m1
8	Htr1a	5-Hydroxytryptamine (serotonin) receptor 1A	Htr1a-Rn00561409_s1
9	Htr2a	5-Hydroxytryptamine (serotonin) receptor 2A	Htr2a-Rn00568473_m1
10	Htr3a	5-Hydroxytryptamine (serotonin) receptor 3a	Htr3a-Rn00577803_m1
11	Htr7	5-Hydroxytryptamine (serotonin) receptor 7	Htr7-Rn00576048_m1
12	Gabra3	Gamma-aminobutyric acid (GABA-A) receptor, alfa 3	Gabra3-Rn00567055_m1
13	Gabrb2	Gamma-aminobutyric acid (GABA-A) receptor, beta 2	Gabrb2-Rn00564149_m1
14	Gabrb3	Gamma-aminobutyric acid (GABA-A) receptor, beta 3	Gabrb3-Rn00567029_m1
15	Gabbr1	Gamma-aminobutyric acid (GABA) B receptor 1	Gabbr1-Rn00578911_m1
16	Gabbr2	Gamma-aminobutyric acid (GABA) B receptor 2	Gabbr2-Rn00582550_m1
17	Gad2	Glutamic acid decarboxylase 2	Gad2-Rn00561244_m1
18	Gad1	Glutamic acid decarboxylase 1	Gad1-Rn00566593_m1
19	Glra1	Gglycine receptor, alpha 1	Glra1-Rn00565582_m1
20	Glra2	Glycine receptor, alpha 2	Glra2-Rn00561280_m1
21	Glra3	Glycine receptor, alpha 3	Glra3-Rn01638847_m1
22	Glrb	Glycine receptor, beta	Glrb-Rn00583966_m1
23	Slc6a5	Glycine transporter 2	Slc6a5-Rn01475607_m1
24	Il6ra	Interleukin 6 receptor, alpha	Il6ra-Rn00566707_m1
25	Il1r1	Interleukin 1 receptor 1	Il1r1-Rn00565482_m1
26	Cx3cl1	Chemokine (C-X3-C) ligand 1	Cx3cl1-Rn00593186_m1
27	Cx3cr1	Chemokine (C-X3-C) receptor 1	Cx3cr1-Rn00591798_m1
28	Cxcl12	Chemokine (C-X-C motif) ligand 12	Cxcl12-Rn00573260_m1
29	Cxcr4	Chemokine (C-X-C motif) receptor 4	Cxcr4-Rn00573522_s1
30	Ccl2	Chemokine (C-C motif) ligand 2	Ccl2-Rn00580555_m1
31	Slc12a5	Potassium-chloride cotransporter 2	Slc12a5-Rn00592624_m1
32	Slc12a2	Sodium-potassium-chloride cotransporter member 1	Slc12a2-Rn00582505_m1
33	Hcn1	Hyperpolarization activated cyclic nucleotide-gated channel 1	Hcn1-Rn00584498_m1
34	Hcn2	Hyperpolarization activated cyclic nucleotide-gated channel 2	Hcn2-Rn01408575_gH
35	Hcn3	Hyperpolarization activated cyclic nucleotide-gated channel 3	Hcn3-Rn00586666_m1
36	Hcn4	Hyperpolarization activated cyclic nucleotide-gated channel 4	Hcn3-Rn00586666_m1
37	Scn9a	Voltage-gated Na channel, 9	Scn9a-Rn00591020_m1
38	Scn10a	Voltage-gated Na channel, 10	Scn10a-Rn00568393_m1
39	NAPE-PLD	N-acil-phoszphatidylethanolamine-hydrolising phospholipase D	NAPE-PLD-Rn01786262_m1
40	Faah	Fatty acid amid hydrolase	Faah-Rn00577086_m1
41	Ntrk2	Neurotrophic tyrosine kinase, receptor, 2	Ntrk2-Rn00820626_m1
42	Bdnf	Brain derived neurotrophic factor	Bdnf-Rn00560868_m1
43	Pkcg	Protein kinase C, gamma	Prkcc-Rn00440861_m1
44	Mapk8	Mitogene-activated protein kinase 8 (JNK)	Mapk8-Rn01453358_m1
45	GAPDH	Glyceraldehyde-3-phosphate dehydrogenase	Gapdh-Rn99999916_s1
46	ARBP	Acidic ribosomal phosphoprotein P0	Arbp-Rn00821065_g1
47	PPIA	Peptidylprolyl isomerase A	Ppia-Rn00690933_m1
		Mandatory control gene:	
48	18S rRNA	18S ribosomal RNA	18S-Hs99999901_s1

The gene expression values have been calculated according to the 2^−ΔΔCt^ comparative threshold cycle method [52]. First, the threshold cycle (Ct), indicating the cycle number by which the amount of the amplified target reaches a fixed threshold, was defined for all investigated genes (Table 1). Then, the Ct of each gene has been normalized (ΔCt) to the average Ct of the three endogenous housekeeping genes (GAPDH, ARBP, PPIA) according to the formula: ΔCt = Ct (target gene) − Ct (housekeeping genes). The ΔCt values were calculated from tissue samples obtained from both the control and CFA-injected animals. Making a relative comparison between PCR signals of the target gene transcripts in tissue samples obtained from the CFA-injected animals to that obtained from the control animals, ΔΔCt values were calculated according to the following equation: ΔΔCt = ΔCt (treated samples) − ΔCt (control samples). Finally, obtaining a relative quantitative value proportional to the changes of the RNA levels evoked by CFA injections, the final value of 2^−ΔΔCt^ (fold change over the control level) [52] was calculated. Statistical significance of the changes was calculated according to the ANOVA statistical probe.

### Western blot analysis

The changes in the quantity of IL-1R1 protein during the first 3 days after CFA injection were measured by Western blot analysis. The spinal cord tissue sample collection procedure was the same as that described at the TLDA analysis. Tissue samples were sonicated in 20 mM Tris (pH 7.4) lysis buffer supplemented with protease inhibitors (4 mM EDTA, 2.5 mM EGTA, 2 nM PMSF, 26 μM benzamidine, 8 μM pepstatine A, 2 μg/ml soybean trypsine inhibitor, 2 μg/ml leupeptine, 2 μg/ml aprotinin). The cell debris were removed by centrifugation (10 min at 1500 rcf and 4 °C), then the supernatant was again centrifuged (20 min at 12,000 rcf and 4 °C). The pellet was re-suspended in lysis buffer containing 1% Triton X-100 and 0.1% SDS. The samples were stored at −70 °C until use.

The protein concentration of the samples was determined using the detergent-compatible BCA assay (Pierce, Rockford, USA). The samples were dissolved in reducing sample buffer (50 μg protein/lane) and run on 10% SDS-polyacrylamide gels [53]. The separated proteins were electrophoretically transferred onto polyvinilidene difluoride (PVDF) membrane (Millipore, Bedford, USA). The membranes were blocked with 10% bovine serum albumin (Sigma, St Louis, MO, USA) in Tween/Tris-buffered salt solution (TTBS) solution (20 mM TRIS, 500 mM NaCl, pH 7.5, 0.05% Tween-20) for 2 h at room temperature and then were incubated with anti-IL-1R1 receptor antibody raised in goat (1:500, RnD Systems, Minneapolis, USA, catalog no.: AF-771) overnight at 4 °C. After extensive washing with TTBS, the blots were incubated with a rabbit-anti-goat IgG secondary antibody conjugated with HRP (1:1000 DakoCytomation, Glostrup, Denmark catalog no.: P0448). The immunostained protein bands were visualized with a diaminobenzidine chromogen reaction.

For quantitative analysis, the blots were also probed for a loading control of β-tubulin. The blots were treated first with a mouse-anti-β-tubulin antibody (1:2000 dilution, Sigma, catalog no.: T8328) followed by a rabbit-anti-mouse IgG conjugated with HRP (1:500 dilution, DakoCytomation, catalog no.: P0447) secondary antibody. The immunostaining was developed by an amino-ethyl carbasole substrate kit (Vector, Burlingame, CA, USA, catalog no.: SK4200). Quantification of the immunoblots was performed using Image J software. The optical density values obtained for IL-1R1 were normalized to density values acquired for β-tubulin. The Western blot analysis and the calculation of the relative quantity of IL-1R1 receptor protein were conducted on three independent preparations. From quantitative data, mean and SEM values were calculated. Data obtained from control and CFA-injected samples were compared using a one-way ANOVA statistical probe.

### Immunohistochemistry

#### Tissue preparation

Immunohistochemical experiments were performed on three controls and three CFA-injected adult male Wistar rats (250–300 g) 3 days after CFA injection. The animals were deeply anesthetized with sodium pentobarbital (50 mg/kg i.p.) and transcardially perfused first with Tyrode’s solution oxygenated with a mixture of 95% 0_2_ and 5% CO_2_, followed by a fixative containing 4% paraformaldehyde in 0.1 M phosphate buffer (PB; pH 7.4). The L4 lumbar segment of the spinal cord was removed from all animals, post-fixed in the same fixative overnight, and immersed in 10 and 20% sucrose dissolved in 0.1 M PB until they sank. After freeze-thawing in liquid nitrogen, 50-μm-thick serial sections were cut on a Vibratome.

#### Single immunoperoxidase staining

Free-floating sections were first incubated with an antibody raised against IL-1R1 in goat (diluted 1:100; RnD Systems) for 2 days at 4 °C. The sections were then transferred into biotinylated rabbit anti-goat IgG (1:200, Vector Laboratories, Burlingame, CA, USA, catalog no.: PK-4001) for 5–6 h. Thereafter, they were treated with an avidin-biotinylated horseradish peroxidase complex (diluted 1:100, Vector Laboratories, Burlingame, CA, USA, catalog no.: PK-4001) and the immunoreaction was completed with a diaminobenzidine chromogen reaction. Before the antibody treatments, the sections were kept in 20% normal rabbit serum (Vector Laboratories, Burlingame, CA, USA, catalog no.: Z0819) for 50 min. The antibodies were diluted in 10 mM Tris phosphate-buffered isotonic saline (TPBS; pH 7.4) to which 1% normal rabbit serum (Vector Laboratories, Burlingame, CA, USA) was added. Sections were mounted on glass slides and covered with DPX neutral medium.

#### Controls

**Fig. 1:**
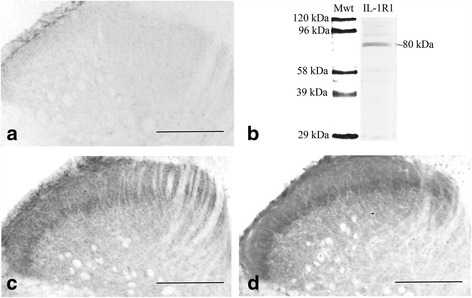
Specificity of the anti-IL-1R1 antibody and distribution of IL-1R1 immunoreactivity in the spinal dorsal horn. **a** Adsorption of anti-IL-1R1 antibody to recombinant IL-1R1 peptide completely abolished the immunostaining. **b** Western blot analysis reinforces the specificity of the anti-IL-1R1 antibody. The single immunoreactive band indicates that the antibody detects a protein with a molecular mass of ~80 kDa that corresponds to the molecular weight of IL-1R1. **c**, **d** Micrographs showing immunoreactivity for IL-1R1 in control (**c**) and CFA-injected rats (**d**) 3 days after CFA-injection. Bars 100 μm

As a part of the immunohistochemical protocol, we tested the specificity of the primary antibody on tissue sections by treating the diluted anti-IL-1R1 with recombinant rat IL-1R1 protein (Sino Biological Inc, Beijing, China, catalog no.: 80028R08H50) for the purpose of antibody adsorption. The recombinant IL-1R1 peptide was mixed with the antibody (1 μg peptide per 1 μg antibody), left at 4 °C for 16–18 h and then centrifuged. Free-floating sections were incubated according to the single immunoperoxidase staining protocol by using the antibody against IL-1R1 treated with the IL-1R1 peptide as primary serum. Under these conditions, specific immunostaining was completely abolished (Fig. 1a).

To obtain a more global view about the specificity of the anti-IL-1R1 antibody, a Western blot analysis was performed as described in the section “Western blot analysis.” The immunostaining of the blot revealed only one immunoreactive band at molecular weight of 80 kDa (Fig. 1b) corresponding to the molecular weight of IL-1R1 [36].

To check the specificity of the immunostaining method, sections were treated by the immunocytochemical procedure as described earlier, with the primary antibody omitted or replaced with normal rabbit serum (diluted 1:100). Under these conditions, no peroxidase reaction was observed.

#### Double immunofluorescent staining

A double-immunostaining protocol was performed to study the co-localization of IL-1R1 with various neuronal and glial markers. Free-floating sections were first incubated with a mixture of antibodies that contained goat anti-IL-1R1 (diluted 1:100, R&D Systems) and one of the following antibodies: guinea-pig anti-calcitonin gene-related peptide (CGRP; diluted 1:2000, Peninsula Labs,, San Carlos, CA, USA, catalog no.: T-5027), biotinylated isolectin B4 (IB4; diluted 1:2000, Sigma, catalog no.: 121414), guinea-pig anti-vesicular glutamate transporter 2 (VGLUT2; diluted 1:2000, Chemicon, Temecula, CA, USA, catalog no.: AB2251), mouse anti-vesicular gamma-amino butyric acid transpofter (VGAT; diluted 1:200, Synaptic Systems, Goettingen, Germany, catalog no.: 131011), mouse anti-glial fibrillary acidic protein (GFAP; diluted 1:1000, Chemicon, catalog no.: MAB3402), mouse anti-CD11b (1:500, Bachem Holding, Bubendorf, Switzerland, catalog no.: T-3102), rabbit anti-potassium-chloride co-transporter 2 (KCC2; 1:2000, Upstate Biotechnology Inc Lake Placid NY New York, catalog no.: 07-432), mouse anti-postsynaptic density protein 95 (PSD-95; 1:100, Frontier, Geumcheon-Gu, Seoul, Korea, catalog no.: AB2723), or mouse anti-gephyrin (diluted 1:100, Synaptic Systems, catalog no.: 147021) for 2 days at 4 °C. The sections were then transferred for an overnight treatment into the mixture of donkey anti-goat IgG conjugated with Alexa Fluor 555 (diluted 1:1000, Molecular Probes, Eugene, OR, USA, catalog no.: A21432), and donkey anti-guinea-pig IgG conjugated with Alexa Fluor 488 (diluted 1:1000, Molecular Probes, Life Technologies catalog no.: A11055), streptavidin conjugated with Alexa Fluor 488 (diluted 1:1000, Molecular Probes, catalog no.: S11223), donkey anti-mouse conjugated with Alexa Fluor 488 (diluted 1:1000, Molecular Probes, catalog no.: A31570), or donkey anti-rabbit conjugated with Alexa Fluor 488 (diluted 1:1000, Molecular Probes, catalog no.: A21206) for 5–6 h at room temperature. Before the antibody treatments, the sections were kept in 10% normal donkey serum (Vector Labs., Burlingame, California, USA, S30-100ML) for 50 min. Antibodies were diluted in 10 mM TPBS (pH 7.4) to which 1% normal donkey serum (Vector Labs., Burlingame, California, USA) was added. Sections were mounted on glass slides and covered with Vectashield mounting medium (Vector Labs., Burlingame, California, USA, H-1000).

#### Confocal microscopy and quantitative analysis

Single 1-μm-thick optical sections were scanned with an Olympus FV1000 confocal microscope. Scanning was carried out with a ×40 oil-immersion lens (NA 1.3). The confocal settings (laser power, confocal aperture and gain) were identical for all means, and care was taken to ensure that no pixels corresponding to puncta immunostained for IL-1R1 and the other markers were saturated. The scanned images were processed by Adobe Photoshop CS3 software. By filtering the background staining out with a high-pass intensity filter, threshold values were set for both IL-1R1 and the other markers. The co-localization of IL-1R1 with neuronal and glial markers was quantitatively analyzed in the double-stained sections. A 10 × 10 standard square grid with an edge-length of 4 μm was put onto confocal images obtained from 1-μm-thick single optical sections. IL-1R1-immunostained spots over the edges of the standard grid were counted in laminae I and II. The selected IL-1R1-immunostained spots were then examined to determine whether they were located within the confines of areas immunoreactive for the axonal (CGRP, IB4-binding, VGLUT2, VGAT), glial (GFAP, CD11b), and postsynaptic membrane (PSD95, gephyrin) markers. In case of sections double-stained for IL-1R1 and KCC2, the selected IL-1R1-immunostained spots were checked to define whether they were aligned along KKC2-immunostained membranes (considered as somatodendritic membrane expression of IL-1R1) or located within areas surrounded by KCC2-immunostained membranes (considered as cytoplasmic expression of IL-1R1 at the somatodendritic compartment of neurons). Three randomly selected confocal sections were analyzed from each animal. Thus, the calculation of quantitative data, mean values, and SEM, was based on the investigation of nine sections. Data obtained from control and CFA-injected animals were compared. Statistical differences between the two experimental groups were computed using a Mann-Whitney test.

## Results

### Complete Freund adjuvant-evoked inflammation as a model of inflammatory pain

We intended to explore the expression pattern of IL-1R1 in the superficial spinal dorsal horn both in naïve animals and in inflammatory pain. Since the complete Freund adjuvant (CFA)-evoked inflammatory pain model has already been established in our laboratory [54], we selected this pain model for our experiments. As we predicted it from our previous studies [54], subcutaneous injection of CFA into the right hind paw evoked a strong inflammation and the development of a prominent mechanical allodynia.

**Fig. 2:**
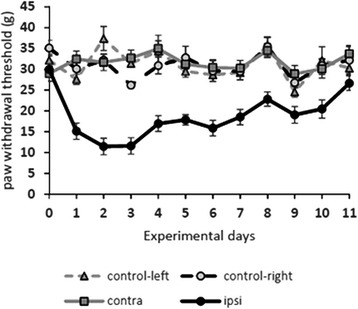
Mechanical withdrawal threshold of rats during the course of CFA-induced inflammation of the hind paw. The histogram shows the mechanical withdrawal threshold (MWT) on both hind limbs of control animals and animals receiving CFA injection into the right hind paw. Note that MWT values appeared to be remarkably stable throughout the entire length of the experimental period in control animals and in the untreated left hind paw of animals receiving CFA injection into the right hind paw. However, CFA injection resulted in a substantial drop in MWT values in the right hind paw of experimental animals which peaked at post-injection days 2 and 3 and returned back to normal values by post-injection day 11. Data are shown as mean ± SEM

The mechanical withdrawal thresholds (MWT) of the untreated control animals and those of the non-treated left hind paw of experimental animals were very similar in all animals throughout the entire duration of the experiment (Fig. 2). The CFA injection resulted in a sudden drop in MWT of the treated right hind paw. The decline in MWT values peaked at post-injection days 2 and 3. From post-injection day 4, MWT values showed a continuous elevation and they returned back to the control values by post-injection day 11 (Fig. 2).

### Overexpression of IL-1R1 in CFA-evoked inflammatory pain

Although the expression of IL-1R1 has already been demonstrated in the spinal cord [43, 46], there has been no attempt to follow the potential changes in the expression of the receptor in inflammatory pain. Thus, we intended to explore how the expression of the receptor changes in CFA-induced inflammatory pain at the level of both mRNA and protein, and how the changes in the quantities of the receptor protein can be visualized with the aid of immunohistochemical staining which is essential for the cellular localization of the receptor.

To find out how IL-1R1 receptor mRNA level changes in the spinal dorsal horn, a TILDA essay was performed. Comparing the mRNA levels of tissue samples obtained from the dorsal horn of control animals and from the dorsal horn of CFA-injected animals ipsilateral to the CFA injection at post-injection day 3 (at the peak of mechanical allodynia), we observed a highly significant, approximately six times increase in the IL-1R1 mRNA (*p* = 0.0002) in the experimental animals (Table 2).

**Table 2:** List of genes that showed statistically significant changes in tissue samples obtained from the spinal dorsal horn of CFA-injected animals ipsilateral to the CFA injection at post-injection day 3 (at the peak of mechanical allodynia)

Gene symbol	Gene name	ΔC_t_ control	ΔC_t_ treated	Fold change 2^-ΔΔCt^	*p* value
Faah	fatty acid amid hydrolase	6.4 ± 0.01	6.7 ± 0.1	0.78	0.0368
Gria2	ionotropic glutamate receptor, AMPA2	3.0 ± 0.24	3.3 ± 0.06	0.77	0.0447
il1r1	interleukin 1 receptor 1	14.7 ± 0.001	12.1 ± 0.002	6.02	0.0002
Nape-pld	N-acyl-phosphatidylethanolamine-hydrolising phospholipase D	7.0 ± 0.06	6.9 ± 0.17	1.11	0.0016

Values obtained from CFA-injected animals were compared to values obtained from control animals. Note that we observed a highly significant, approximately six times increase in the quantity of IL-1R1 receptor mRNA in the experimental animals

To define whether CFA injection-evoked changes in the IL-1R1 mRNA expression were confined only to the ipsi-lateral spinal cord, we performed the TILDA assay also on tissue samples obtained from the dorsal horn of CFA-injected animals contra-lateral to the CFA injection. We found that although at a bit lower extent and with higher inter-animal variations, the expression of IL-1R1 mRNA also showed an elevation in the contra-lateral dorsal horn, indicating that the increase in IL-1R1 mRNA is not confined to the regions of the spinal gray matter receiving sensory innervations from the inflamed area of the skin, but the local paw inflammation may evoke a more general effect on spinal pain processing. Although this finding was very exciting, we intended to focus the present study to the dorsal horn ipsilateral to the CFA-evoked inflammation. For this reason, the rest of the experiments had been conducted exclusively on spinal cord samples obtained from the dorsal horn ipsilateral to the CFA injection.

**Fig. 3:**
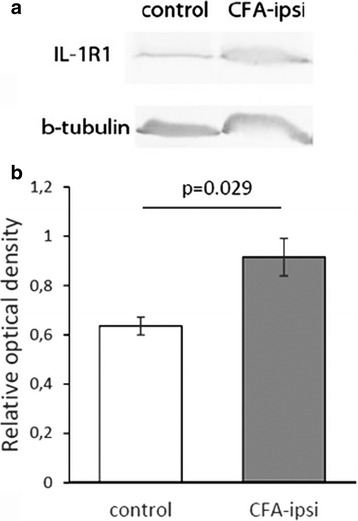
CFA-evoked inflammation of the hindpaw initiates an overproduction of IL-1R1 protein in the spinal dorsal horn of rats. **a** Representative immune-blots showing immunoreactive bands for IL-1R1 and β-tubulin (loading control) in Western blots of tissue samples obtained from the L3–L5 lumbar segments of the spinal dorsal horn of the control and CFA-injected animals at post-injection day 3. **b** Histogram showing the optical densities of IL-1R1 immunostained bands (see on insert **a**) calculated in proportion to the optical densities of β-tubulin (loading control) immunostained bands (see on insert **a**). IL-1R1 protein level was found to be significantly higher than the control value (*p* = 0.029). Data are shown as mean ± SEM

To demonstrate this increase also at the level of the receptor protein, we carried out Western blot analysis on tissue samples obtained from the dorsal horn of the control animals and from the dorsal horn of CFA-injected animals ipsilateral to the CFA injection at post-injection day 3 (Fig. 3a). A densitometric analysis showed an approximately 1.5-fold increase in the IL-1R1 protein level (*p* = 0.029) in the experimental animals (Fig. 3b).

Concerning the distribution of IL-1R1 in the spinal dorsal horn, peroxidase-based single immunostaining revealed a punctuate immunostaining in the dorsal horn (Fig. 1c). The staining within laminae I–II was a bit stronger than that in deeper laminae. The heaviest staining was confined to the inner lamina II, which appeared as a densely immunostained band traversing the entire medio-lateral extent of the dorsal horn (Fig. 1c). CFA-evoked inflammation induced an increase in the density of the immunostaining throughout the entire dorsal horn including laminae I–II ipsilateral to the injection site (Fig. 1d). The immunostaining in the inner lamina II also became more abundant (Fig. 1d).

### Mechanical and thermal allodynia does not develop in the lack of IL-1R1

The substantial increase in IL-1R1 expression in the spinal dorsal horn evoked by CFA injection into the hind paw seems to be in full agreement with earlier observations showing that IL-1β is also upregulated in inflammatory pain [4, 7–10]. In addition to IL-1β–IL-1R1 signaling, however, there are a number of other factors that also contribute to the development of hyperalgesia and allodynia in inflammatory pain (for review see McMahon and Koltzenburg, 2006) [55]. Thus, we intended to explore how much is the contribution of the IL-1R1-mediated signaling events to the development of mechanical and thermal allodynia in inflammatory pain.

**Fig. 4:**
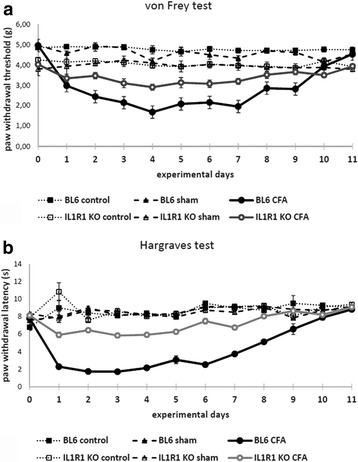
Mechanical withdrawal threshold and thermal withdrawal latency of wild type and IL-1R1 knockout mice during the course of CFA-induced inflammation of the hind paw. **a** The histogram shows the mechanical withdrawal threshold (MWT) of wild type (BL6) and IL-1R1 knockout (IL-1R1 KO) mice receiving different treatments in the three experimental groups: group (1) complete Freund-adjuvant (CFA) injection (BL6 CFA, IL-1R1 KO CFA); group (2) physiological saline injection (BL6 sham, IL-1R1 KO sham); and group (3) without any treatment (BL6 control, IL-1R1 KO control). Measurements were made only on the right hind paw which received the physiological saline or CFA injections. **b** The histogram shows the thermal withdrawal latency (TWL) of wild type (BL6) and IL-1R1 knockout (IL-1R1 KO) mice receiving different treatments in the three experimental groups: group (1) complet Freund-adjuvant (CFA) injection (BL6 CFA, IL-1R1 KO CFA); group (2) physiological salt solution injection (BL6 sham, IL-1R1 KO sham); and group (3) without any treatment (BL6 control, IL-1R1 KO control). Measurements were made only on the right hind paw which received the physiological saline or CFA injections. Data are shown as mean ± SEM

We injected CFA into the right hind paw of wild type and IL-1R1 knockout (B6.12957-IL1r1 ^tm1/mx^/J, stock #:003245) mice and followed the development of mechanical and thermal allodynia by measuring the mechanical withdrawal threshold (MWT) and thermal withdrawal latency (TWL) values daily from the day of injection till post-injection day 11. Instead of CFA, some animals received an injection of physiological saline (sham-injected animals). Comparing the MWT values measured in untreated and sham-injected animals to those observed in IL-1R1 knockout mice, we found that the IL-1R1 knockout animals showed a higher sensitivity for acute mechanical stimulation than the naïve animals. The MWT values of the untreated control and those of the IL-1R1-depleted mice were very stable throughout the entire duration of the experiment (Fig. 4a). The values varied between 4.1–5.0 g with an average of 4.81 ± 0.29 g and between 3.0–5.0 g with an average of 3.94 ± 0.51 g in case of the wild type and the IL-1R1 knockout animals, respectively (Fig. 4a). The difference between the basal values of the wild type and knockout animals turned out to be highly significant (*p* = 0.0000002). Injection of physiological saline into the right hind paw did not cause any detectable change in MWT values (Fig. 4a). The CFA injection, however, resulted in a sudden drop in MWT of the treated right hind paw of the wild type animals. The decline peaked at post-injection day 4 when MWT values dropped to 1.68 ± 0.06. Then, MWT values showed a continuous elevation and they returned back to the control values by post-injection days 10–11 (Fig. 4). The IL-1R1 knockout animals showed only a slight decline in MWT values to CFA injection, and even this moderate decline lasted only for a week after CFA injection (Fig. 4a). The degree of decrease in the MWT values reached the statistically significant level only between post-injection days 1–7 in the IL-1R1 knockout animals (*p* = 0.00004–0.006). However, the difference between MWT values measured after the injection of CFA in wild type and IL-1R1 knockout animals were highly significant from post-injection day 1 till post-injection day 9 (*p* = 0.000004–0.01).

The course and degree of thermal allodynia evoked by CFA injection was similar to that of mechanical allodynia both in the wild type and IL-1R1 knockout animals. The basal TWL values, however, did not show any difference between the wild type and IL-1R1 knockout animals. The TWL values were very stable throughout the entire duration of the experiment and varied between 4.9 and 10.2 s with an average of 7.47 ± 1.22 s, and between 6.3 and 10.0 s with an average of 8.15 ± 0.85 s in case of the wild type and the IL-1R1 knockout animals, respectively (Fig. 4b). Injection of physiological saline into the right hind paw did not cause any detectable change in TWL values (Fig. 4b). The CFA injection, however, resulted in a sudden drop in TWL of the treated right hind paw of the wild type animals. The decline peaked at post-injection days 2 and 3 when TWL values dropped to 1.74 ± 0.1 s and 1.72 ± 0.08 s, respectively. Then, TWL values showed a continuous elevation, and they returned back to the control values by post-injection days 9–10 (Fig. 4b). The IL-1R1 knockout animals showed only a slight decline in TWL values to CFA injection, and even this moderate decline lasted only for a week after CFA injection (Fig. 4a). The degree of decrease in the TWL values reached the statistically significant level only between post-injection days 1–7 in the IL-1R1 knockout animals (*p* = 0.0005–0.029). However, the difference between TWL values measured after the injection of CFA in wild type and IL-1R1 knockout animals was highly significant from post-injection day 1 till post-injection day 9 (*p* = 0.000003–0.021).

### IL-1R1 is expressed on both neurons and glial cells

The robust difference in the degree of mechanical and thermal sensitivity evoked by CFA-induced inflammation in wild type and IL-1R1 knockout animals indicates that IL-1R1-mediated signaling play a major role in the development of inflammatory pain. For the proper interpretation of the mechanism of IL-1R1 activation, however, it is necessary to obtain a proper knowledge about the expression pattern of the receptor on neurons and glial cells. It has already been reported that IL-1R1 is expressed on both neurons [8, 9, 13, 40] and glial cells [13, 28, 42–46], but nothing is known about the relative distribution of the receptor on these two distinct cell populations. Because of the conflict in the presently available interpretations of neuronal and glial contribution to the net effect of IL-1R1-mediated signaling events on the activities of neuronal assemblies [45, 47–49], we intended to explore the proportional distribution of IL-1R1 on neurons and glial cells. Thus, we performed double immunostainings in which we investigated the localization of IL-1R1 immunoreactive spots on neurons and glial cells immunostained for markers specific for various axon terminals, perikarya and dendrites of neurons, postsynaptic membranes of excitatory, and inhibitory synaptic contacts, as well as astrocytes and microglial cells.

#### Localization of IL-1R1 on axon terminals

To study the expression of IL-1R1 on central axon terminals of peptidergic and non-peptidergic nociceptive primary afferents as well as axon terminals of putative glutamatergic and GABAergic spinal neurons, we investigated the co-localization of the receptor with calcitonin gene-related peptide (CGRP) and isolectin B4 (IB4)-binding as well as vesicular glutamate transporter type 2 (VGLUT2) and vesicular gamma amino butyric acid transporter (VGAT), markers selectively labeling axon terminals that we intended to identify [56–60].

**Fig. 5:**
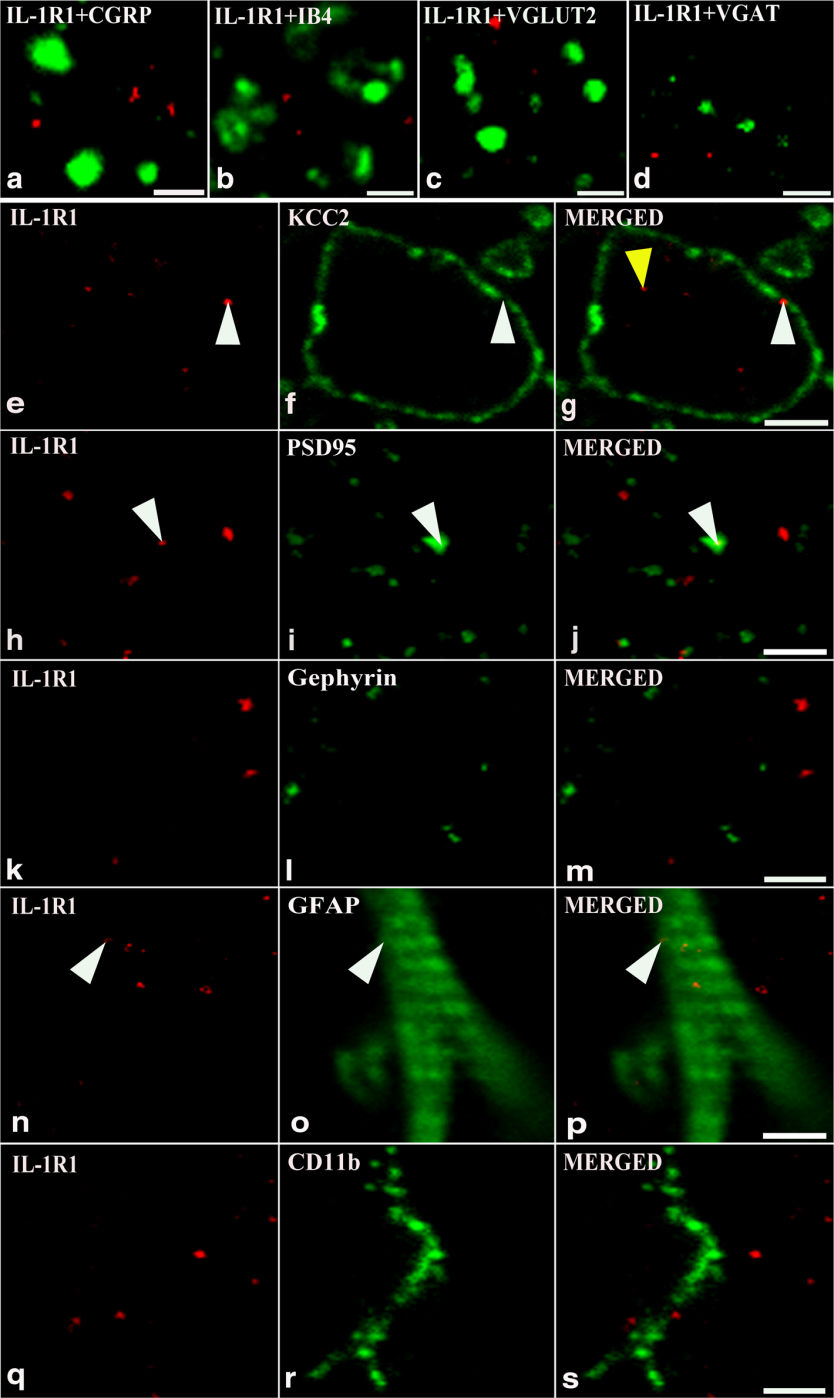
Localization of IL-1R1 on neurons and glial cells in the superficial spinal dorsal horn of rats. Micrographs of single 1-μmthick laser scanning confocal optical sections illustrating the co-localization between immunolabeling for IL-1R1 (*red*; **a**–**d**, **e**, **h**, **k**, **n**, **q**) and immunoreactivity for markers that are specific for axon terminals of peptidergic (CGRP, *green*; **a**) and non-peptidergic (IB4 binding, *green*; **b**) primary afferents, axon terminals of excitatory (VGLUT2, *green*; **c**) and inhibitory (VGAT, *green*; **d**) intrinsic neurons, astrocytes (GFAP, *green*; **o**) and microglial cells (CD11b, *green*; **r**), and postsynaptic membranes of excitarory (PSD95, *green*; **i**) and inhibitory (gephyrin, *green*; **l**) synapses in the superficial spinal dorsal horn. Mixed colors (*yellow*; marked by *white arrowheads*) on the superimposed images (**j**, **m**, **p**, **s**) indicate *double-labeled structures*. The absence of *yellow color* on **a**–**d** indicates a lack of IL-1R1 expression on axon terminals of various origin. IL-1R1 immunoreactive spots appear in two different localization on the micrographs showing immunostaining also for KCC2 (*green*, **f**, **g**): (1) They can be aligned along the lines defined by the KCC2 immunostaining (cell membrane localization; *white arrowheads* on **f** and **g**). (2) They can also be located in areas surrounded by the KCC2-immunostained cell membranes (cytoplasmic localization, *yellow arrowhead* on **g**. Bars 2 μm (**a**–**d**), 5 μm (**n**–**s**), and 10 μm (**e**–**m**)

**Fig. 6:**
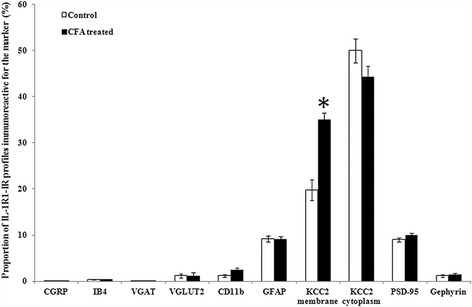
Histogram showing the CFA-evoked inflammation induced changes in the degree of co-localization between immunoreactivity for IL-1R1 and selected neuronal and glial markers in the superficial spinal dorsal horn of rats. *Columns* indicate the percentages of profiles immunoreactive for IL-1R1 that were found to be labeled also for the selected markers, and the ones that were aligned along KCC2 immunoreactive membranes (localization on the somatodendritic membrane of neurons) or were located within areas surrounded by KCC2 immunoreactive membranes (localization within the cytoplasm of the somatodendritic compartment of neurons). *White columns* show data obtained from control animals, whereas *black columns* represent values found in CFA-injected animals 3 days after CFA injection into the right hind paw. *Asterisk* indicate that CFA-evoked inflammation significantly increased the number of spots immunoreactive for IL-1R1 on the somato-denditic membrane of neurons (*p* = 0.000001). Data are shown as mean ± SEM

Confirming results of previous studies [57, 59, 61–66], we have revealed strong punctate immunostainings for CGRP, VGLUT2, and VGAT in laminae I–II, and IB4-binding also labeled a large number of axon terminals in lamina IIi. Despite of the extensive and intense axonal staining, the double-stained specimen showed almost a complete segregation between IL-1R1 and the axonal markers (Figs. 5a–d, 6). From the 1099, 1331, 1333, and 1001 IL-1R1 immunoreactive dots recovered from the nine investigated sections immunostained for CGRP, IB4-binding, VGLUT2 or VGAT only 1 (0.1 ± 0.01%), 4 (0.3 ± 0.10%), 16 (1.2 ± 0.46%) ,and 1 (0.1 ± 0.04%) of IL-1R1 immunoreactive puncta were recovered on axon terminals immunoreactive for CGRP, binding IB-4, and immunoreactve for VGLUT2 and VGAT, respectively.

#### Localization of IL-1R1 on the somatodendritic membrane of neurons

From the members of the cation-chloride cotransporter family which are primarily responsible for the regulation of intracellular chloride concentration of cells [67–71] the potassium-chloride co-transporter 2 (KCC2) has been shown to be expressed only on neurons [67, 72–76]. It appears to be primarily responsible for the Cl-mediated hyperpolarizing postsynaptic currents evoked by GABAergic and glycinergic transmission [70, 76–79], and its extensive expression has convincingly been demonstrated on the somatodendritic membranes but not on axon terminals of spinal dorsal horn neurons [80–82].

Thus, to study the expression of IL-1R1 on the somatodendritic compartment of neurons, we labeled the somatodendritic cell membrane with an immunostaining against KCC2 and was looking for IL-1R1 immunoreactive spots which were either aligned along lines defined by the KCC2 immunostaining (localization in the cell membrane) or located in areas surrounded by the KCC2-immunostained cell membranes (localization in the cytoplasm). After careful evaluation of the double-stained sections, from the 1332 IL-1R1 immunoreactive spots recovered from the nine investigated sections immunostained for KCC2, we found 264 (19.8 ± 2.27%) within the somatodendritic membranes whereas 665 (49.9 ± 2.56%) of them were recovered in the cytoplasm of neurons (Figs. 5e–g, 6).

#### Localization of IL-1R1 in postsynaptic densities of putative excitatory and inhibitory synapses

Co-precipitation studies of Gardoni et al. [41] suggested that IL-1R1 distributed along the somatodendritic membranes of hippocampal neurons are enriched in postsynaptic membranes of excitatory synapses. Since we found that approximately 20% of the IL-1R1 immunoreactive spots were scattered along the somatodendritic membrane, we intended to test what proportions of the receptor molecules can be inserted into the postsynaptic membranes of putative excitatory and inhibitory synapses. Thus, we were looking for IL-1R1 immunoreactive spots within the confines of PSD95, a postsynaptic density protein characteristic for glutamatergic synapses [83–85] and gephyrin, a scaffolding protein associated with GABA_A_ and glycine receptors [86–88], which are immunoreactive postsynaptic membranes of putative excitatory and inhibitory synapses, respectively.

In agreement with the report of Gardoni et al. [41], we revealed a remarkable enrichment of IL-1R1 immunoreacitvity in some postsynaptic membranes of putative excitatory synapses. After the evaluation of the nine double-stained sections, we recovered 1122 PSD95 immunoreactive spots, putative postsynaptic membranes of excitatory synapses, and found that 72 (6.4 ± 0.43%) of them were also stained for IL-1R1. Most interestingly, we recovered 117 IL-1R1 immunoreactive dots within the confines of PSD95 immunoreactive postsynaptic membranes representing 9.04 ± 0.43% of the total number of IL-1R1 immunoreactive spots (Figs. 5h–j and 6) which is about half of the IL-1R1-immunostained spots on the somato-dendtiric membrane of neurons. The colocalization between gephyrin and IL-1R1 immunostaining was much lower. From the 1250 gephyrine immunoreactive postsynaptic membranes counted in the sections, only 15 (1,2 ± 1.1%) was also stained for IL-1R1, and the 26 IL-1R1 immunoreactive dots recovered within the confines of gephyrin immunoreactive postsynaptic membranes represented only 2.17 ± 0.3% of the total number IL-1R1 immunoreactive spots (Figs. 5k–m and 6) which is about 10% of the IL-1R1-immunostained spots on the somato-dendtiric membrane of neurons.

#### Localization of IL-1R1 on astrocytes and microglial cells

To study the expression of IL-1R1 on astrocytes and microglial cells, we investigated the co-localization of the receptor with glial fibrillary acidic protein (GFAP) and CD11b, markers that selectively label astrocytes and microglial cells, respectively.

Evaluating the sections double-stained for IL-1R1 and one of the glial markers we found, a low level of co-localization with GFAP and almost a complete segregation between Il-1R1 and CD11b. From the 1350 and1330 IL-1R1 immunoreactive dots recovered from the nine investigated sections immunostained for GFAP and CD11b, only 125 (9.3 ± 0.65%) and 16 (1.2 ± 0.30%) of IL-1R1 immunoreactive puncta were recovered on astrocytes and microglial cells immunoreactive for GFAP and CD11b, respectively (Figs. 5n–s, and 6).

### Overexpression of IL-1R1 on extrasynaptic somatodendritic membrane compartments of neuron in CFA-evoked inflammatory pain

We found a robust expression of IL-1R1 on the somatodendritic compartment of neurons, an enrichment of the receptor in the postsynaptic membranes of some excitatory synapses and only a moderate one on astrocytes in control animals. As we also demonstrated, inflammatory pain evoked by CFA injection into the hind paw increased the expression IL-1R1 in a substantial extent 3 days after the CFA injection, at the time of the summit of hyperalgesia and allodynia. CFA-evoked inflammatory pain, however, may modify not only the quantity but also the distribution pattern of IL-1R1 in the superficial spinal dorsal horn. Thus, we intended to explore how the expression pattern observed in the control animals may change in CFA-evoked inflammatory pain condition. For this reason, we repeated the complete set of immunocytochemical double staining experiments on sections obtained from CFA-injected experimental animals 3 days after the CFA injection.

The proportion of IL-1R1 immunoreactive spots revealed on axon terminals and glial cells were found to be very similar to the control values (Fig. 6); although, concerning the increase in the quantity of IL-1R1, the unchanged proportions may indicate a slight increase in the total number of IL-1R1 both on axon terminals and glial cells. In case of the somatodendritic compartment of neurons, the proportion of IL-1R1 immunoreactive spots did not show any change in the cytoplasm and on postsynaptic membranes immunoreactive for PSD95 and gephyrin either; although similarly to the axonal and glial expression, the unchanged proportions may indicate a slight increase in the total number of IL-1R1 in postsynaptic membranes of both excitatory and inhibitory synapses. The only obvious change in the distribution of IL-1R1 immunoreactivity that we observed at the level of statistical significance was an increase in the proportion of IL-1R1-immunostained dots that were aligned along the lines defined by KCC2 immunostaining, indicating a robust increase in the expression of IL-1R1 on the somatodendritic membrane compartment of neurons. In this case, the 19.8 ± 2.27% value of IL-1R1 immunoreactive spots which were aligned along the lines defined by the KCC2 immunostaining in control animals increased to 35.1 ± 1.5% (*p* = 0.002) (Fig. 6).

## Discussion

Investigating the expression and cellular distribution of IL-1R1 in the superficial spinal dorsal horn of rats in control animals and also in CFA-evoked inflammatory pain, we found a highly significant, approximately six times increase in the IL-1R1 mRNA and a 1.5 times increase in the IL-1R1 protein levels in the dorsal horn of experimental animals ipsilateral to CFA injection on post-injection day 3, at the time of the summit of the mechanical and thermal sensitivity of the animals. Underlying the importance of IL-1R1-mediated signaling in the development of inflammatory pain, here we also demonstrated that in the lack of IL-1R1, the development of both mechanical and thermal allodynia evoked by CFA injection into the hind paw becomes inhibited at a considerable extent. Studying the cellular distribution of IL-1R1 in rats, we found a robust expression of IL-1R1 on the somatodendritic compartment of neurons and an enrichment of the receptor in the postsynaptic membranes of some excitatory synapses. In contrast to the abundant neuronal localization, we observed only a moderate expression of IL-1R1 on astrocytes and almost nothing on microglial cells. CFA injection into the hindpaw caused a remarkable increase in the expression of IL-1R1 on the somatodendritic membrane of neurons, but did not alter the glial expression of the receptor.

### IL-1R1-mediated signaling mechanisms play a major role in spinal pain processing

It is well established that the pro-inflammatory cytokine IL-1β facilitates the transmission and processing of noxious inputs at the spinal level [4, 6]. Further support to this idea is provided by studies showing that intrathecal administration of IL-1β in healthy rodents induces hyperalgesia and allodynia [16–21, 89] and enhance both the acute response to C-fiber stimulation and the wind-up activity of dorsal horn neurons [15, 22]. IL-1β is reported to be upregulated in the spinal cord during inflammatory [4, 6, 7, 23] and neuropathic pain [11, 12] which led to the notion that IL-1β can be an important contributor to the development of persistent pain states [7, 9, 21, 28]. All known biological functions of IL-1β are mediated by IL-1R1 [34]. IL-1R1 antagonist (IL-1Ra) completely abolish IL-1β-induced enhancement of nociceptive neuron responses [90] and produce anti-allodynic effects in rat model of neuropathic [91, 92], inflammatory [9], and cancer pain [8, 13, 21].

Along this line of evidence, here we showed that not only IL-1β but also IL-1R1 mRNA as well as protein levels also became substantially upregulated in the spinal dorsal horn in CFA-evoked inflammatory pain condition. This is a quite remarkable novel finding since the increased expression of IL-1R1 is likely to be necessary for the enhancement of the effects of IL-1β in persistent pain conditions. The increased expression of IL-1R1 and the consecutive enhancement of the Il-1β–IL-1R1 signaling mechanisms seem to be one of the most important contributors to the development of spinal central sensitization and the following chronic inflammatory pain condition, since in the lack of the receptor the development of both mechanical and thermal allodynia evoked by CFA injection into the hind paw becomes inhibited at a considerable extent, as we showed here. This notion is strongly reinforced by the finding of Wolf et al. [93] reporting that neuropathic pain was also markedly reduced with the deletion of IL-1R1 or transgenic overexpression of IL-1Ra.

### Neuronal expression of IL-1R1 dominates over glial localization in the superficial spinal dorsal horn

According to the prevailing view, the effects of IL-1β on spinal pain processing are mediated by direct actions on neurons [47–49] through its specific receptor [9, 28, 30, 40]. Consistent with this notion, we recovered more than two-thirds of IL-1R1 immunostaining on the somatodendritic compartment of neurons in control rats. Concerning the concept of direct action of IL-1β on neurons, it is even more remarkable that the CFA-evoked inflammatory pain condition further increased the somatodendritic expression of IL-1R1. Three days after CFA injection into the hind-paw, approximately 85% of IL-1R1 immunostaining was observed in the somatodendritic compartment of neurons. Even more importantly, this increase was mostly due to the abundant expression of IL-1R1 on the somatodendritic membrane.

The activation of neuronal IL-1R1 initiates phosphorylation of NR1 and/or NR2B subunits of NMDA receptors [9, 28] contributing to the trafficking of NMDA receptors to the cell membrane [94]. The insertion of additional NMDA receptors into postsynaptic membranes enhances NMDA receptor-mediated inward currents and increases intracellular Ca^2+^ concentration [26, 27, 30] leading to the facilitation of nociceptive C-fiber stimulation [21, 44, 95, 96], wind-up phenomena [97], post-discharge of wind-dynamic range neurons in the spinal dorsal horn [15, 21], and consecutively to the development of LTP, central sensitization, and pain [98]. Our present results indicate that synaptic NMDA receptors and glutamatergic synaptic transmission can be influenced by IL-1R1 located both in the postsynaptic and extrasynaptic membranes. In agreement with the findings of Gardoni et al. [41], we found that in control rats, nearly half of the IL-1R1 immunostaining recovered on the somatodendritic membrane of neurons was co-localized with PSD95, one of the postsynaptic density protein of glutamatergic synapses [83–85]. However, this enrichment of IL-1R1 was found only in a minor fraction of glutamatergic synapses. Only 6–7% of PSD95-immunostained spots were also stained for IL-1R1. If we assume that a larger population of glutamatergic synapses can be modulated by the activation of IL-1R1, as it is indicated by the aforementioned functional studies, the activation of extrasynaptic IL-1R1 should be considered also effective concerning the potentiation of NMDA receptors similarly to the synaptic ones. This notion is strongly supported by another finding of the present experiments, showing that the relative proportion of IL-1R1 expression 3 days after CFA injection into the hind paw nearly doubled on the somatodentritic membrane but the co-localization of the receptor with PSD95 was found to be unchanged.

Here we also demonstrated that in addition to excitatory synapses, IL-1R1 was also located in postsynaptic membranes of inhibitory synapses. We found that approximately 10% of IL-1R1 immunostaining on the somatodendritic membrane of neurons was co-localized with gephyrin, a scaffolding protein within the postsynaptic membrane of inhibitory synapses [86–88]. In the light of the report of Chirila et al. [99], showing that in the superficial spinal dorsal horn glycinergic synapses on inhibitory GABAergic, neurons exhibit LTP, occurring rapidly after exposure to the inflammatory cytokine IL-1β, it is likely that the gephyrin-immunostained spots found to be positive also for IL-1R1 in the present study represent glycinergic synapses. Although we observed IL-1R1 immunostaining only in approximately 1% of gephyrin-immunostained spots, one can regard this finding functionally significant, since the potentiation of glycinergic synapses on GABAergic neurons evoked by in vitro bath application of IL-1β [21, 100] or peripheral inflammation in vivo [99] could evoke considerable disinhibition in pain processing spinal neural networks [21, 101], an important mechanism that is increasingly appreciated for the generation of chronic pain [102, 103].

Although the prevailing view assumes that IL-1β is directly acting on neurons, some authors claim that the synaptic effects of IL-1β are not mediated directly via activation of neuronal IL-1R1, but rather, indirectly via IL-1R1 being expressed on glial cells in the superficial spinal dorsal horn [43, 45, 46, 104]. In support of this hypothesis, Gruber-Schoffnegger et al. [45] reported that in the presence of fluorocitrate, an inhibitor of glial metabolism, IL-1β depressed, rather than potentiated, NMDA receptor-mediated currents, suggesting that IL-1β needs to activate glia to facilitate glutamatergic transmission at C-fiber synapses. According to this alternative assumption, activation of glial IL-1R1 by IL-1β leads to the release of gliotransmitters which may amplify glutamatergic synaptic transmission. Our present results, although emphasizes the direct neuronal action of Il-1β, does not provide any evidence against the possibility of the action of IL-1β on glial IL-1R1. In the present study, we found that nearly 10% of IL-1R1 immunostaining was localized on astrocytes, but we were not able to detect any substantial immunolabeling on microglial cells.

## Conclusions

In conclusion, we may speculate that due to their activation by peripheral inflammation nociceptive primary afferents release ATP in the spinal dorsal horn [105]. ATP acting on glial adenosine receptors [106] initiates IL-1β release from astrocytes [8–10, 45]. IL-1β binds to neuronal IL-1R1, the activation of which amplifies NMDA receptor-mediated synaptic currents [9, 28] and potentiates glycinergic synapses on GABAergic neurons [99], thus reducing inhibitory synaptic transmission. In addition, IL-1β also interacts with IL-1R1 expressed by astrocytes [45]. In response to this, astrocytes release gliotransmitters [50, 51] which may further facilitate glutamatergic transmission. These diverse signaling cascades acting in concert with each other can substantially contribute to the enhancement of the excitation level of spinal pain processing neural networks leading to the development of spinal central sensitization and consecutive pain.

## Abbreviations

AMPA: Alpha-amino-3-hydroxy-5-methyl-4-isoxazolepropionic acid
BCA: Bicinchoninic acid
cAMP: Cyclic adenosine monophosphate
CFA: Complete Freund adjuvant
HRP: Horseradish peroxidase
IL1-R: Interleukin-1 receptor
IL-1β: Interleukin-1béta
LTP: Long-term potentiation
MWT: Mechanical withdrawal threshold
NMDA: N-methyl-D-aspartate
NR2B: N-methyl-D-aspartate receptor subtype 2B
PCR: Polymerase chain reaction
PSD: Postsynaptic density
PVDF: Polyvinilidene difluoride
SDS: Sodium dodecyl sulfate
TLDA: TaqMan low-density array
TTBS: Tween/tris-buffered salt solution
TWL: Thermal withdrawal latency
